# An update on aging and dementia in Chile

**DOI:** 10.1590/S1980-57642014DN84000003

**Published:** 2014

**Authors:** Patricio Fuentes, Cecilia Albala

**Affiliations:** 1 Geriatrics Section, Medicine Department, Clinical University Hospital of Chile and Cognitive Neurology and Dementia Unit, Neurology Service, Hospital del Salvador, Santiago, Chile; 2 Institute of Nutrition and Food Technology, Santiago, Chile El Líbano 5524, Macul, Santiago,Chile.

**Keywords:** demography, epidemiology, dementia, aging, Chile

## Abstract

Chile is in an advanced demographic transition stage with the population over 60 years of age representing 15% of the total population and whose number of elderly has more than doubled between 1990 and 2014. Rapid economic advancement has promoted significant changes in social organization to which the country is not accustomed. The mental health problems of the elderly are particularly challenging to the country's present social and health structures. The prevalence of dementia in people over 60 years exceeds 8% and is even higher in the rural population. There is more training on dementia in the local medical and scientific community, increased awareness within the civilian community but insufficient responsiveness from the state to the broad diagnostic and therapeutic requirements of patients and caregivers. The objective of the present study was to provide an update of the information on dementia in the context of the ageing process in Chile.

## Abstract (pt)

O Chile está num avançado estágio de transição epidemiológica, com a população de 60 anos ou mais representando 15% da população total e o número de idosos tem mais que dobrado entre 1990 e 2014. O rápido avanço econômico tem promovido significantes mudanças na organização social para as quais o país não está habituado. Os problemas de saúde mental em idosos são particularmente desafiadores para as estruturas sociais e de saúde atuais do país. A prevalência de demência entre aqueles com idade superior a 60 anos excede 8% e é mais elevada na população rural. Há mais treinamento em demência na população médica e comunidade científica locais, aumentando o conhecimento dentro da comunidade civil, porém, com insuficiente resposta do estado às necessidades diagnósticas e terapêuticas de pacientes e cuidadores. O objetivo deste estudo foi providenciar uma atualização da informação sobre demência no contexto de envelhecimento cerebral do Chile.

## INTRODUCTION

In recent decades, Latin American countries have experienced rapid demographic and epidemiological transition,^1,2^ which has led to a rapidly aging population and increase in the frequency of chronic and degenerative diseases. In the regional context, Chile is the country whose life expectancy at birth (LEB) has grown fastest. Between the periods 1970-75 and 2010-2015 LEB increased from 60.5 to 76.5 years in men and from 66.8 to 81.7 years in women.^3,4^ It is estimated that the magnitude of the increase in life expectancy seen in Chile, together with the sustained decline in fertility, currently at 1.9, is set to cause high sustained growth of the older population.^5^

In Chile, the absolute number of elderly has more than doubled between 1990, when the population >60 years was 1179,637 people and 2014, with 2,679,910 people in this age group. In relative terms, the proportion of people >60 years has increased from 9% of the total population in 1990 to 14.9% in 2014.^3,4^ The age group growing fastest is the 80 years and over bracket whose annual rate of growth in Latin America and the Caribbean has attained 3.94%.^6^ This age group in Chile has grown by 130% between 1990 and 2010 (Table 1).

**Table 1:** Human development changes in Chile.

	1990	2000	2012
Human Development Index	0.698	0.748	0.819
GDP (US$ PPP)	6583	10470	18419
Q5/Q1	16.9	17.5	14.1
% Urban Population	83	85.6	88
Mean Years of Schooling	8.1	8.8	9.7
Population ≥60 years	9	10.2	13.5
Infant Mortality	16	9	8
Mortality in under 5s	21	11	9
Global fertility rate	2.6	2	1.9
Overall life expectancy at birth	73.7	77	79
Men	69	75	78
Women	76	81	82
Life expectancy at 60 years	19	21	23

GDP: gross domestic product; PPP: purchasing power parity; Q1/Q5: quintile ratio.

The socioeconomic and health achievements of the country place Chile among the high-income countries, with a GDP of US$ 21000 (IMF 2014) and health indicators comparable with western developed countries and the USA. However, persistent major inequalities in wealth distribution persist with a significant impact on health indicators of the elderly.^7,8^ The objective of this review was to update the information on the problem of dementia in Chile, in the context of rapid population aging.

Dementia, characterized by a progressive deterioration in intellectual function affecting functionality, is probably the most feared and devastating disease among the problems affecting the elderly, representing a major cause of disability and dependence in older age.

According to the 10/66 dementia research group, dementia is the leading cause of dependency in older people.^9,10^ The mean proportion of dependency attributable to dementia is 36%, followed by limb paralysis/weakness with 11.9% and stroke with 8.7%. In Chile, the burden of disease study performed in 2007^11^ identified dementia as the third leading cause of DALYs lost in the 60-74y age group (25,531 DALYs lost), after cataracts (28,350) and Ischemic Heart disease (26,506), and the second leading cause of DALYs lost in women.

The 2013 PAHO/WHO estimated prevalence of dementia for Latin America (standardized by European region population) was 8.5%^12^ and WHO estimates for the high-income countries in the Southern cone of Latin America (Argentina, Chile and Uruguay) predicts a 77% increase in cases of dementia by 2030 and 134-146% for the rest of Latin America. The most common form of dementia is Alzheimer's disease (AD) constituting 60 to 75% of dementias in the elderly.^13,14^

The first available population data in Chile is derived from the WHO Age associated dementias study conducted in Concepción, Chile in 1990-92^15^ where a crude prevalence of 4.38 (3.57-5.33) was reported.^16,17^ In the cited study, Alzheimer's disease was the most common type of dementia, accounting for 85% of the cases. The latest population data available about dementias in the country and its contribution to the burden of disease in the elderly in Chile is from the National Survey of Dependency (NSD) in the elderly. The NSD was conducted in 2010 in a representative sample of 4860 people 60y and older.^18^ Briefly, the NSD study was a cross-sectional survey, carried out in a probabilistic sample of independent-living older adults, 60 years and older, residents of all Chilean Regions. A stratified, multi-stage sampling design, with selection proportional to population size, was the method applied, ensuring the participation of people from both urban and rural areas. From within each municipality, sectors (and subsequently households) were selected randomly. The population of 80 years and older was oversampled to allow for a more precise estimation of dependency in this growing sector of the Chilean population. The study was approved by the Institute of Nutrition and Food Technology, University of Chile Ethics Committee. Dementia was defined using the test validated in the WHO Age associated dementias study in Concepción, consisting of a score<22 on the Mini-Mental State Examination (MMSE)^19^ and a score>5 on the Pfeffer activities questionnaire^20^ with sensitivity of 94.4% (95%CI: 70.6-99.7) and specificity of 83.3% (95%CI: 72.3-90.7).^21^ Symptoms of Depression were evaluated with the Geriatric Depression Scale (GDS-15)^22^ adopting a cut point >4 as positive screening.

The total crude prevalence of dementia in people 60y and older observed was 7.0% (women 7.7%; men 5.9%; p=0.15) and proved higher in rural than in urban samples (10.3% vs 6.3%; p=0.002 (Table 2).

**Table 2:** Prevalence (%) of dementia by age group, in urban and rural samples and in men and women.

Dementia	60-64y	65-69y	70-74y	75-79y	80-84y	≥85y
Urban	0.94	3.9	3	8.4	17.2	29
Rural	2.6	5.1	6.9	10.6	29.7	50.4
Total	1.2	4.1	3.7	8.8	19.4	32.6
Men	1	5.9	3.6	6	18.2	24.4
Women	1.4	3.1	3.8	10.1	20	36.5

In both men and women, there is a significant increase in the prevalence of dementia with increasing age (p<0.0001). The same situation was observed in both urban and rural samples (p<0.002).

**Figure 1:**
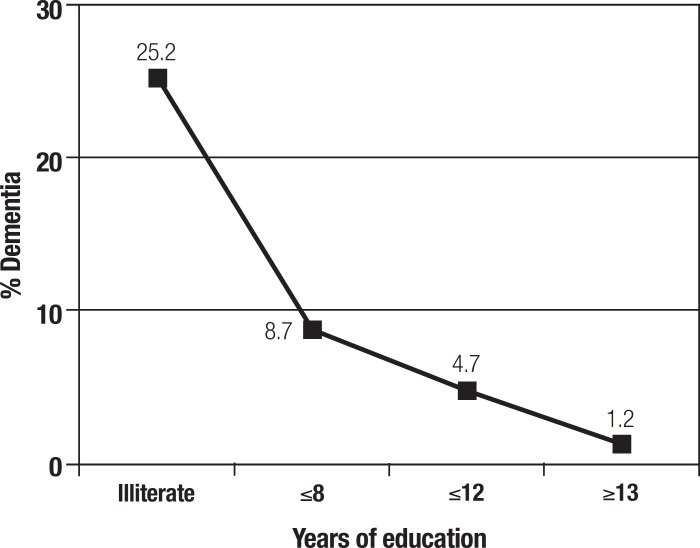
Prevalence of dementia by years of education, Chile 2010.

Figure 1 shows a strong negative association of dementia with years of education (p<0.001). Dementia prevalence in illiterates is 25.2% while the rate in subjects with >13 years of schooling is only 1.2%.

Table 3 shows the proportion of dependence attributable to dementia by gender and area of residence. Around one third of dependency is attributable to dementia, except among men from urban areas where the percentage is lower (27%).

**Table 3:** Dementia, dependence and proportion of dependence attributable to dementia by gender and area of residence.

	Urban %	Rural %	Total %
Dementia	7.3	11.8	8.1
Dependence	19.5	31.0	21.5
Proportion of dependence attributable to Dementia	37.6	38.1	37.8
***Pearson: Design-based F(1. 617) P < 0.001			
Men			
Dementia	5.0	12.8	6.7
Dependence	16.3	33.0	19.8
Proportion of dependence attributable to Dementia	30.7	38.8	33.8
***Pearson: Design-based F(1.868) P < 0.001			
Women			
Dementia	8.6	11.0	9.0
Dependence	21.2	29.6	22.5
Proportion of dependence attributable to Dementia	40.6	37.2	40.0

The adjusted model for dementia showed no association with gender but an independent positive association with rurality and age as well as a negative association with educational level attained (Table 4). The strongest association was observed for age with an OR=10.75 for the 80y and older group in comparison with the 60-69 years-old group, followed by education (OR=2.76) in the group with less than 8 y of schooling compared to the group with 8 y or more.

**Table 4:** Logistic regression model for dementia with years of education, age, gender and rurality as independent variables.

Dementia	OR	95% CI	p
≥8 years of education	1	Reference	
< 8 years schooling*	2.76	1.54-4.94	<0.001
Age 60-69 y	1	Reference	
Age 70-79y	2.56	1.32-4.98	<0.005
Age ≥80y	10.95	6.42-18.7	<0.001
Urban	1	Reference	
Rural	1.42	1.00-2.01	0.045
Men	1	Reference	
Women	1.17	0.77-1.79	0.460

In a subsample of 287 people (50% women) of Mapuche ethnicity, a higher crude prevalence of dementia was found than in individuals of non-Mapuche origin (10.9% vs 6.9%, p<0.02). This association persisted after adjusting for age, sex and educational level (OR=1.6;95% CI 1.06-2.44) as shown in Table 5. However, these results should be interpreted with caution considering that the diagnostic instruments used have not been validated in the Mapuche people.

**Table 5:** Logistic regression model for association of dementia with Mapuche ethnicity adjusted by years of education, age, gender and rurality.

Dementia	Model 1				Model 2		
	OR	95% CI	p		OR	95% CI	P
Mapuche ethnicity	1.60	1.06-2.44	0.027		1.57	1.01-2.43	0.045
Age	1.14	1.11-1.17	<0.001		1.13	1.10-1.16	<0.001
Men	1	reference			1	reference	
Women	1.23	0.82-1.84	0.323		1.15	0.76-1.75	0.507
Urban	1	reference			1	reference	
Rural	1.77	1.27-2.45	0.001		1.41	1.01-1.99	0.047
≥8 years of education					1	reference	
< 8 years of education					2.73	1.52-4.90	0.001

**Figure 2:**
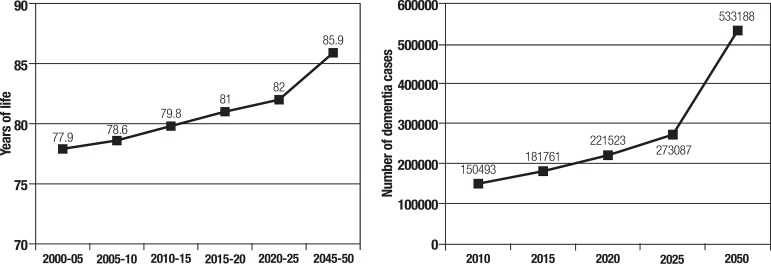
Life expectancy at birth and estimated number of dementia cases. Chile 2002-2050

Based on the prevalence of dementia observed in the present study, the population estimates from the National Institute of Statistics (INE 2014), and considering the estimated increase in life expectancy (United Nations 2010) over the same period, the estimated number of people with dementia was 150,293 in 2010, 181,761 by 2015 reaching 533,188 by 2050 (Figure 2). The estimated number of dementia cases was determined considering the prevalences of dementia by age group found in this survey and in the population at large.

**Progress to date**. Currently, there is no national strategy in Chile for dementia while existing governmental health programmes, such as the National Program for the Elderly or the Mental Health Program, have devoted scant attention to initiatives aimed at the healthcare of persons with dementia. Recent governments have shown no greater sensitivity to the problem and have given it only secondary priority. However, a new workgroup is now in place within the Ministry of Health with the aim of creating a future National Dementia Plan. In 2002, the government established an organization called SENAMA (Servicio Nacional del Adulto Mayor) tasked with improving the quality of life for older people, taking a gerontological perspective; this institution has supported public dementia-related initiatives such as the first Day Care Center for people with dementia in the country, named Kintun,^24^ located in the municipality of Peñalolen in Santiago intended for families of limited means.

Only a few private clinics or large hospitals, concentrated in major cities, have integral units for the diagnosis and treatment of dementia with multidisciplinary professional teams. At the primary care level, there are basic standards and clinical guidelines for cognitive impairment and dementia: a screening instrument called EFAM (Functional Assessment of the Elderly) is in routine use, validated for use in the local setting, which allows the detection of older adults in the community who are at risk of losing functionality in the short and medium term and enables categorization by level of independence.^23^ Furthermore, since 2008 the Preventive Medical Examination for the Elderly (EMPAM) has been in place, as part of the so-called GES guarantee or AUGE (80 pathologies included): this provides a comprehensive benefits package, free and guaranteed to be delivered both in the public and private health systems; it prioritizes certain diseases, but has not yet incorporated dementia despite its recognized disease burden.

At higher levels of health complexity, there are memory clinics in public hospitals, where patients with cognitive disorders can be examined adequately and a probable diagnosis reached within a reasonable timeframe. However, therapeutic options are limited since it is not possible to prescribe approved anti-dementia medication as a result of absence of central funding. Neither are cognitive stimulation programmes readily available, although there are many memory workshops at the county level for cognitively healthy individuals. Appropriate psychotropic drugs for the management of neuropsychiatric symptoms are equally difficult to prescribe under the public system. In general, people with dementia experiencing significant behavioural change have poor access to inpatient care in public hospitals. There are virtually no health structures that can be classed as acute psychogeriatrics units, and the only public hospital in the country specializing in older adults, the Geriatrics National Institute, has major limitations in physical and human resources.

It is noteworthy that the diagnosis and treatment of patients with dementia is mainly carried out by neurologists and geriatricians and only a very small proportion are diagnosed by psychiatrists. Additionally, in recent years there has been little interest in the study and management of mental disorders in the elderly within specialist training, despite the obvious increase in prevalence of these diseases.

There are about 1700 long-term care homes; half of them concentrated in the capital and a third informal.^25^ Most are private and others, run by religious or philanthropic organizations, receive some support from the state. It is clear that the majority of patients with advanced dementia are being cared for by relatives. Day hospitals and day-care centers for patients with cognitive disorders are scarce.

Chile has only three or four research centres in neuroscience applied to neurodegenerative diseases (the University of Chile, the Catholic University and the University of Concepcion) able to compete internationally. Moreover, in the last decade, the country has become an important platform in Latin America for conducting clinical trials with new anti-dementia drugs at various stages of development. A significant contribution regarding training, management and care is provided by the relevant scientific societies and the local association of family volunteers (Corporación Alzheimer Chile). The Society of Neurology, Psychiatry and Neurosurgery of Chile as well as the Society of Geriatrics and Gerontology of Chile collaborate in various workgroups and provide training and information to professionals and community representatives through courses, publications and media campaigns highlighting the enormous challenges Chilean society is set to face in the near future. For 20 years or so, the Corporation Alzheimer Chile, affiliated with Alzheimer's Disease International, has provided education, medical care and psychological support to families and caregivers, particularly those on low incomes.

There is growing interest in incorporating psychogeriatric training on cognitive impairment and dementia into under and postgraduate curricula of health professions in most academic institutions, both public and private. In the short-term, this is expected to reverse the current low level of training of both professionals and non-professionals providing mental health care to older adults.

Chile is facing an ageing population and consequently a steep increase in the incidence and prevalence of dementia, a problem typical of developed countries. However, the dementia challenge is being faced by a system applying standards more typically encountered in developing countries. Socioeconomic inequality and the structure and dynamics of the country's healthcare system prevent delivery of quality care to the majority of those affected by dementia.^8^ Hence, even small improvements in the various agencies and systems will lead to important redress in the medium term.

**Current key needs**. The increasing health problems in aging societies pose large potential demand for health services and expose Ministries of health in Latin America to a significant increase in the health-care budget. The enormous cost of the disease is a challenge for health systems given the predicted increase in prevalence. The accelerated aging of the Chilean population, particularly the group of 80y and older, and the growing number of cases of dementia reported poses a huge challenge to the country. It is imperative to anticipate the growing and urgent demand for a particularly vulnerable group often forgotten and discriminated.

People with dementia live for many years after the first symptoms of the disease. With appropriate support many of them can and should be able to continue participating and contributing to society and to have a good quality of life. In this context, early detection of the disease and the design of programmes aimed at combatting reversible risk factors while stimulating protective factors should be a priority for the public health policies in the country.

A particularly concerning issue is the lack of specialists in mental health for the elderly. Therefore, the training at different levels of complexity for all health workers who work, or will work, with these patients is a matter of national priority. Also, qualified staff and investment in infrastructure and technology will be vital to achieve earlier diagnosis and more effective future therapeutic interventions.

Optimum coordination of social and support networks leveraging the existing community will also be crucial for families and caregivers. All these interventions of urgent implementation must be synchronized with a change in social culture toward greater inclusion of the frail and reducing stigmas.

Finally, interventions aimed at caring for people with dementia are required urgently. Although the proportion of elders living alone remains low (15%), the changing patterns of the population structure will impact living arrangements in the future, with a substantial reduction in family size and consequently, in the availability of family caregivers. Dementia is stressful for caregivers, who need proper support from the financial, legal, social and health systems.

In the NSD, a high burden of care was associated with dementia of the care-recipient and low social support to the caregiver. The high burden of caregivers is a cause of concern. When caregiving is performed under conditions of poverty, without breaks, training and resources for care along with a lack of institutional or social support, there is a high risk of associated morbidity as a result of caregivers missing out on preventive health programmes (mammogram, pap smear, etc.), increased depression rates and decreased immunity. The risk of neglect and abuse is also increased with an overwhelmed caregiver.

The process of population ageing in developing countries has important economic and social consequences. Our societies are getting older faster than developed nations, but in a context of poverty, unequal economic distribution and gender inequality. To face these challenges, timely and integral social policies for pensions, housing and healthcare are required.
